# Autochthonous transmission of *Trichophyton indotineae* through sexual contact, France, 2024

**DOI:** 10.2807/1560-7917.ES.2025.30.26.2500416

**Published:** 2025-07-03

**Authors:** Arnaud Jabet, Thibault Chiarabini, Christophe Hennequin, Fabrice Bouscarat, Nadia Valin, Romuald Cruchet, Nicolas Boo, Johan Chanal, Christine Schuttler, Myriam Kirstetter, Anne-Cécile Normand, Arnaud Fekkar, Valérie Pourcher, Renaud Piarroux, Gentiane Monsel

**Affiliations:** 1 Service de Parasitologie-Mycologie, Hôpital de La Pitié-Salpêtrière, Assistance Publique-Hôpitaux de Paris (AP-HP), Institut Pierre-Louis d’Epidémiologie et de Santé Publique, INSERM, Sorbonne Université, Paris, France; 2 Service de Parasitologie-Mycologie, Hôpital Saint-Antoine, Assistance Publique-Hôpitaux de Paris (AP-HP), Paris, France; 3 Service des Maladies Infectieuses et Tropicales, Hôpital Saint-Antoine, Assistance Publique-Hôpitaux de Paris (AP-HP), Paris, France; 4 Service de Parasitologie-Mycologie, Hôpital Saint-Antoine, AP-HP, Centre de Recherche Saint-Antoine, CRSA, INSERM, Sorbonne Université, Paris, France; 5 Service des Maladies Infectieuses et Tropicales, Hôpital Bichat, Assistance Publique-Hôpitaux de Paris (AP-HP), Paris, France; 6 Centre de Santé Sexuelle Frédéric Edelmann, Hôtel-Dieu, Assistance Publique-Hôpitaux de Paris (AP-HP), Paris, France; 7 Laboratoire Biogroup, Paris, France; 8 Service des Maladies Infectieuses et Tropicales, Hôpital de La Pitié-Salpêtrière, Assistance Publique-Hôpitaux de Paris (AP-HP), Paris, France; 9 Service de Parasitologie-Mycologie, Hôpital de La Pitié-Salpêtrière, Assistance Publique-Hôpitaux de Paris (AP-HP), Paris, France; 10 Service de Parasitologie-Mycologie, Hôpital de La Pitié-Salpêtrière, Assistance Publique-Hôpitaux de Paris (AP-HP), CNRS, Centre d'Immunologie et Des Maladies Infectieuses, Cimi-Paris, INSERM, Sorbonne Université, Paris, France; 11 Service des Maladies Infectieuses et Tropicales, Hôpital de La Pitié-Salpêtrière, Assistance Publique-Hôpitaux de Paris (AP-HP), Institut Pierre-Louis d’Epidémiologie et de Santé Publique, INSERM, Sorbonne Université, Paris, France

**Keywords:** tinea, Trichophyton, sexually transmitted diseases, terbinafine, itraconazole

## Abstract

*Trichophyton indotineae* is an emerging difficult to diagnose dermatophyte species associated with terbinafine resistance, which was initially described in South-Asian and Middle-Eastern countries. We report four cases of sexually transmitted dermatophytosis caused by *T. indotineae* in two female sex-workers, including one transgender woman, and two men who have sex with men. All four cases were acquired in Europe, highlighting the risk of pathogen spread through sexual contact outside initial endemic areas.

*Trichophyton indotineae* is a recently described dermatophyte species associated with several clinical and diagnostic challenges. It is characterised by extensive skin lesions and frequent resistance to terbinafine, reported in up to 75% of isolates. While the rise in difficult-to-treat dermatophytosis cases was initially identified in India in the mid-2010s, infections caused by *T. indotineae* are now being reported across all continents, reflecting a rapid international spread [1]. In addition, increasing attention has recently been directed to the transmission of dermatophytes through sexual activity. Specifically, *Trichophyton mentagrophytes* ITS genotype VII (TMVII) is responsible for severe skin lesions among men who have sex with men (MSM) in European countries [2-4] and in the United States [5]. Here, we report four cases of sexually transmitted dermatophytosis caused by *T. indotineae*, all acquired in Europe, highlighting the risk of local transmission of the pathogen and its potential spread through sexual contact.

## Case descriptions

The four cases were diagnosed in two Parisian tertiary hospitals, in 2024. The main characteristics of the four patients are summarised in the Table. Their age ranged from early 20s to late 60s. Two patients were MSM with multiple sexual partners; the other two were female sex workers, one cisgender and the other transgender. Two patients were living with HIV and received effective antiretroviral therapy. Three patients had not left Europe in the 6–12 months before the onset of lesions. As for the last patient, she had at least not traveled in the 2 months preceding the appearance of lesions and had never been to South Asia or the Middle East. Infections were probably acquired in France for three patients and in Portugal for the fourth. Nonetheless, three patients reported sexual relations with men originating from South Asia in the months before onset. One patient noted inguinal skin lesions on a sexual partner. In all patients, the lesions initially appeared on the buttocks (Figure). For one patient, the lesions subsequently spread to the thighs, pubic area, torso and face.

**Table:** Cases of sexually transmitted, autochthonous *Trichophyton indotineae* infections, France, 2024 (n = 4)

Date of sample	HIV/PrEP	STI history	Country of infection	Treatment delay	Location of the lesions	TRB-resistance *SQLE* sequencing	Prior treatment	Treatment
Jan 2024	HIV	Ct, HPV, Ng, Pp, Tp	France	2 months	Buttocks	No A448T	tS 7 days	oTRB + CPX 1 month, then CPX 1 month
May 2024	HIV	HPV	France	ND	Buttocks	Yes F397L	ND	tKTZ
Aug 2024	PrEP	Tp	Portugal	1 year	Buttocks, thighs, face, torso	No A448T	tS, KTZ, traditional medicines	oTRB 6 weeks
Sep 2024	No	Ng, Pp	France	No	Buttocks	No A448T	ECZ 3 weeks	ECZ then lost to follow-up

CPX: ciclopirox; Ct: *Chlamydia trachomatis*; ECZ: econazole; HPV: human papillomavirus; KTZ: ketoconazole; ND: no data; Ng: *Neisseria gonorrhoeae*; oTRB: oral terbinafine; Pp: *Pthirus pubis*; PrEP: HIV pre-exposure prophylaxis; *SQLE*: squalene epoxidase; STI: sexually transmitted infection; tKTZ: topical ketoconazole; Tp: *Treponema pallidum*; TRB: terbinafine; tS: topical steroids.

**Figure:**
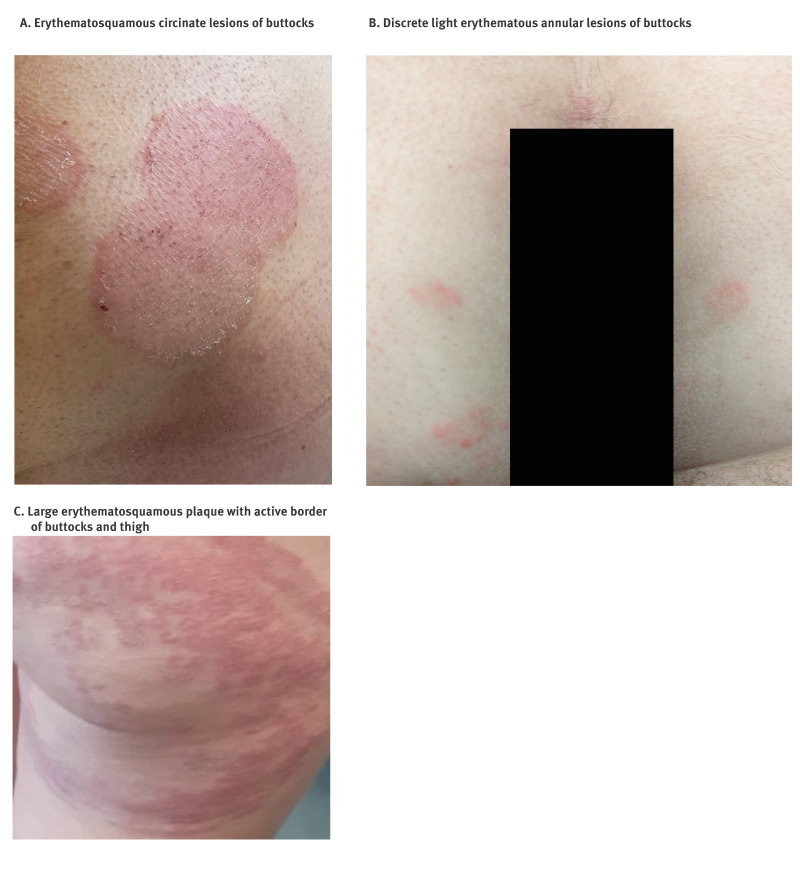
Clinical appearance of *Trichophyton indotineae* infection acquired in Europe through sexual contact, France, 2024 (n = 3)

All isolates were identified as *T. indotineae* using MALDI-TOF mass spectrometry with the MSI-2 database [6]. Diagnosis was further confirmed by sequencing the internal transcribed spacer (ITS) region (GenBank accession numbers: PV760987–PV760990). Using a terbinafine-containing agar method [7], one isolate was found to be resistant to terbinafine. Sequencing of the *SQLE* gene, encoding the squalene epoxidase, revealed in this case a F397L substitution (GenBank accession number: PV788277), which is the most frequent substitution reported in terbinafine-resistant isolates [1]. Otherwise, all the isolates harboured a substitution A448T, which is not associated with terbinafine resistance (GenBank accession numbers: PV788276, PV788278, PV788279) [1].

Two patients received oral terbinafine treatment, whereas one patient (whose isolate was resistant to terbinafine) was treated with topical ketoconazole. In these three cases, complete clinical recovery was achieved, and none of them has experienced a recurrence of the infection to date. One of the two sex workers ceased her activity as a result of the skin lesions. One patient, who had initially been prescribed topical econazole, was lost to follow-up after leaving France.

## Discussion

These four cases are consistent with the diagnosis of sexually transmitted dermatophytosis (STDe) as they include risk factors for sexually transmitted infections (STI) (multiple sexual partners and history of STIs), lesions in the buttocks area and the absence of travel to endemic areas which could have explained the infection. In sexually active patients with tinea corporis, the diagnosis of STDe relies on the presence of STI risk factors and the exclusion of other sources of infection (such as auto-inoculation, zoonotic transmission, etc). The location of the lesions (genital area, buttocks, face) may suggest the diagnosis of STDe, but is not sufficient on its own to confirm it [8]. For the two male patients, TMVII infection had initially been considered as they were MSM. Since 2021, we have identified cases of TMVII infection in Paris among MSM with multiple sexual partners [2,9]. Similar cases were subsequently diagnosed in other French cities, including Lyon [10], but also Strasbourg, Menton, Nice and Grenoble (data not shown). This phenomenon is not limited to France, as similar cases have also been reported in other European countries such as Germany [11], Italy [3], Spain [4], the United Kingdom [12], and Greece [13], as well as in the United States [5]. Healthcare professionals should henceforth also consider *T. indotineae* as a possible diagnosis when evaluating an STDe in an MSM.

Despite close attention to identifying cases in women, all STDe due to TMVII infections in France to date have involved men. Interestingly, we report here two cases of STDe caused by *T. indotineae* in female sex workers. Sexually transmitted dermatophytosis cases have already been reported among sex workers [2,5,14]. While they are vulnerable to STIs themselves, they may also transmit the infection to their clients and play a role in the spread of the pathogen. As contagiousness may persist despite treatment, as exemplified by TMVII, skin-to-skin contact involving lesional areas should be avoided until complete resolution, potentially impacting the professional activity of sex workers [2].

In 2023, the European Centre for Disease Prevention and Control (ECDC) issued an alert regarding the presence of *T. indotineae* in Europe and encouraged its monitoring through the use of EpiPulse [15]. To date, cases have been reported in Belgium, Czechia, Denmark, Estonia, Finland, France, Germany, Greece, Hungary, Ireland, Italy, the Netherlands, Portugal, Spain, Sweden, Switzerland, Türkiye and the United Kingdom [16-26]. Most of the published cases could be linked to exposure in South Asia or the Middle East [16], and limited data were available regarding the few apparently autochthonous cases [27]. However, our four cases strongly indicate autochthonous transmission of *T. indotineae* in Europe. Similarly, autochthonous infections have also recently been documented in Latin America [28] and China [29], supporting the global spread and establishment of *T. indotineae* outside its initial endemic areas. Nonetheless, the fact that three of four patients reported having had sexual intercourse with men from South Asia suggests that the infection may be linked to partners originating directly from endemic areas.

Identification of two types of substitutions by *SQLE* sequencing revealed that more than one clone of *T. indotineae* is circulating through sexual transmission in Europe. In contrast to our cases, the first reported *T. indotineae* infection linked to sexual transmission was associated with exposure in South Asia [30]. The case concerned a woman diagnosed in the United States, who developed lesions on her thighs following sexual intercourse in South Asia with a man presenting with skin lesions. Sexual intercourse could be an underestimated driver of transmission, especially given that tinea cruris lesions are common in *T. indotineae* infections [1]. At our two centres, only one other case had been diagnosed before this, in July 2023, with no identified epidemiological link to South Asia. That patient was a man infected either in France or during travel to Mediterranean countries, and there was no evidence suggesting sexual transmission [31]. 

Mycological sampling is strongly recommended for the proper management of all patients with cutaneous dermatophytosis, as it is becoming increasingly difficult to identify those who may be infected with *T. indotineae* based solely on travel history. Moreover, species-level identification can be challenging due to the morphological similarity of *T. indotineae* to *T. mentagrophytes* and *T. interdigitale*, hindering both clinical management and epidemiological surveillance. While ITS region sequencing remains the gold standard for identifying *T. indotineae*, MALDI-TOF mass spectrometry using the MSI-2 application has demonstrated excellent performance and may serve as a cost-effective and more accessible alternative diagnostic tool, enabling case detection and epidemiological analysis [6,24,26]. 

Since these initial four cases of STDe due to *T. indotineae*, we have identified two additional cases in Belgium, while two other highly suspected cases are currently being investigated in France (data not shown). These cases are part of a prospective binational cohort study on STDe, launched in March 2025. These new cases have reinforced our intention to raise an early alert regarding the concerning cases diagnosed in 2024.

## Conclusion

Medical professionals involved in sexual health, along with service users, should be informed about this emerging issue, associated with therapeutic challenges. Close attention should be given to the potential increase in sexually transmitted cases of *T. indotineae*, which could become a considerable sexual health concern – similar to TMVII – and contribute to the further international spread of *T. indotineae*.

## Data Availability

The sequence data are available on Genbank (PV760987-PV760990, PV788276-PV78829).
